# RREB1-induced upregulation of the lncRNA AGAP2-AS1 regulates the proliferation and migration of pancreatic cancer partly through suppressing ANKRD1 and ANGPTL4

**DOI:** 10.1038/s41419-019-1384-9

**Published:** 2019-02-27

**Authors:** Bingqing Hui, Hao Ji, Yetao Xu, Juan Wang, Zhonghua Ma, Chongguo Zhang, Keming Wang, Yan Zhou

**Affiliations:** 1 Department of Oncology, Second Affiliated Hospital, Nanjing Medical University, Nanjing, 210000 Jiangsu China; 2 The Second Clinical Medical College of Nanjing Medical University, Nanjing, 210000 Jiangsu China; 3 Department of Obstetrics and Gynecology, The First Affiliated Hospital of Nanjing Medical University, Nanjing, 210000 Jiangsu China; 4 The First Clinical Medical College of Nanjing Medical University, Nanjing, 210000 Jiangsu China; 5 Department of Oncology, Yixing Hospital Affiliated to Jiangsu University, Wuxi, 214200 Jiangsu China

## Abstract

Long noncoding RNAs (lncRNAs) have been reported to be involved in a variety of human diseases, including cancers. However, their mechanisms have not yet been fully elucidated. We investigated lncRNA changes that may be associated with pancreatic cancer (PC) by analyzing published microarray data, and identified AGAP2-AS1 as a relatively overexpressed lncRNA in PC tissues. qRT-PCR assays were performed to examine expression levels of AGAP2-AS1. MTT assays, colony formation assays, and EdU assays were used to determine the proliferative capacity of cells. Flow cytometry and TUNEL assays were used to study the regulation of AGAP2-AS1 in the cell cycle and apoptosis. Transwell experiments were used to study changes in cell invasion and metastasis, and a nude mouse model was established to assess the effects of AGAP2-AS1 on tumorigenesis in vivo. RNA sequencing was performed to probe AGAP2-AS1-related pathways. Subcellular fractionation and FISH assays were used to determine the distribution of AGAP2-AS1 in PC cells, and RIP and ChIP were used to determine the molecular mechanism of AGAP2-AS1-mediated regulation of potential target genes. Increased expression of AGAP2-AS1 was associated with tumor size and pathological stage progression in patients with PC. RREB1 was found to activate transcription of AGAP2-AS1 in PC cells. AGAP2-AS1 affected proliferation, apoptosis, cycle arrest, invasion, and metastasis of PC cells in vitro, and AGAP2-AS1 regulated PC proliferation in vivo. Furthermore, AGAP2-AS1 epigenetically inhibited the expression of ANKRD1 and ANGPTL4 by recruiting zeste homolog 2 (EZH2), thereby promoting PC proliferation and metastasis. In summary, our data show that RREB1-induced upregulation of AGAP2-AS1 regulates cell proliferation and migration in PC partly through suppressing ANKRD1 and ANGPTL4 by recruiting EZH2. AGAP2-AS1 represents a potential target for the diagnosis and treatment of PC in the future.

## Introduction

As reported in *Cancer Statistics (2018)*, pancreatic cancer (PC) is the fourth most common cause of cancer-related deaths in the USA and other Western countries, and is predicted to become the second leading cause in 10 years. Its average annual incidence rate in the United States is 12.5 per 100,000 (3% of all cancers), but the mortality rate is very high (10.9 per 100 000, 7% of all cancers)^1^. Given current medical capabilities, it is difficult to diagnose PC at an early stage. The vast majority of PC cases show metastasis at the initial diagnosis; only 9.7% are diagnosed in a localized phase. There are currently no conventional biomarkers that are suitable for use in the diagnosis of PC^1^. Therefore, there is an urgent need to further study the mechanisms underlying PC occurrence and progression, to identify biomarkers with early diagnostic value and to find new and promising therapeutic targets.

Over the past few years, the development of next-generation sequencing techniques and bioinformatics methods has contributed to the completion of many large-scale and multitissue sequencing programs^2–4^. These have revealed that only a small fraction (<2%) of the mammalian genome encodes proteins, while the vast majority (>90%) of a genome is transcribed into noncoding RNAs (ncRNAs) that have limited or no protein-coding potential^5^. Despite initial controversy about their biological properties, there is increasing evidence that ncRNAs have an important regulatory function^6–9^. Long ncRNAs (lncRNA) are a class of ncRNAs with length greater than 200 nucleotides; some studies have demonstrated that lncRNAs are involved in a variety of biological processes, including epigenetic regulation, imprinting, RNA decay, alternative splicing, cell cycle control, cell differentiation, cancer metastasis, and drug resistance^6–9^. In addition, there is increasing evidence that lncRNA expression is abnormally upregulated or downregulated abnormally in many cancers, including PC, and many lncRNAs are associated with cancer metastasis, recurrence, and poor prognosis^10–15^. Therefore, lncRNAs have become an important focus in cancer research, and there is an urgent need to solve key questions regarding the systematic identification and characterization of PC-associated candidate lncRNAs.

This study demonstrates that understanding the functional linkages and potential molecular mechanisms involved in PC, particularly those relating to AGAP2-AS1, will help to elucidate the important role of lncRNAs in gene regulation and tumorigenesis.

## Results

### AGAP2-AS1 expression is upregulated in PC and is associated with poor prognosis

**Fig. 1:**
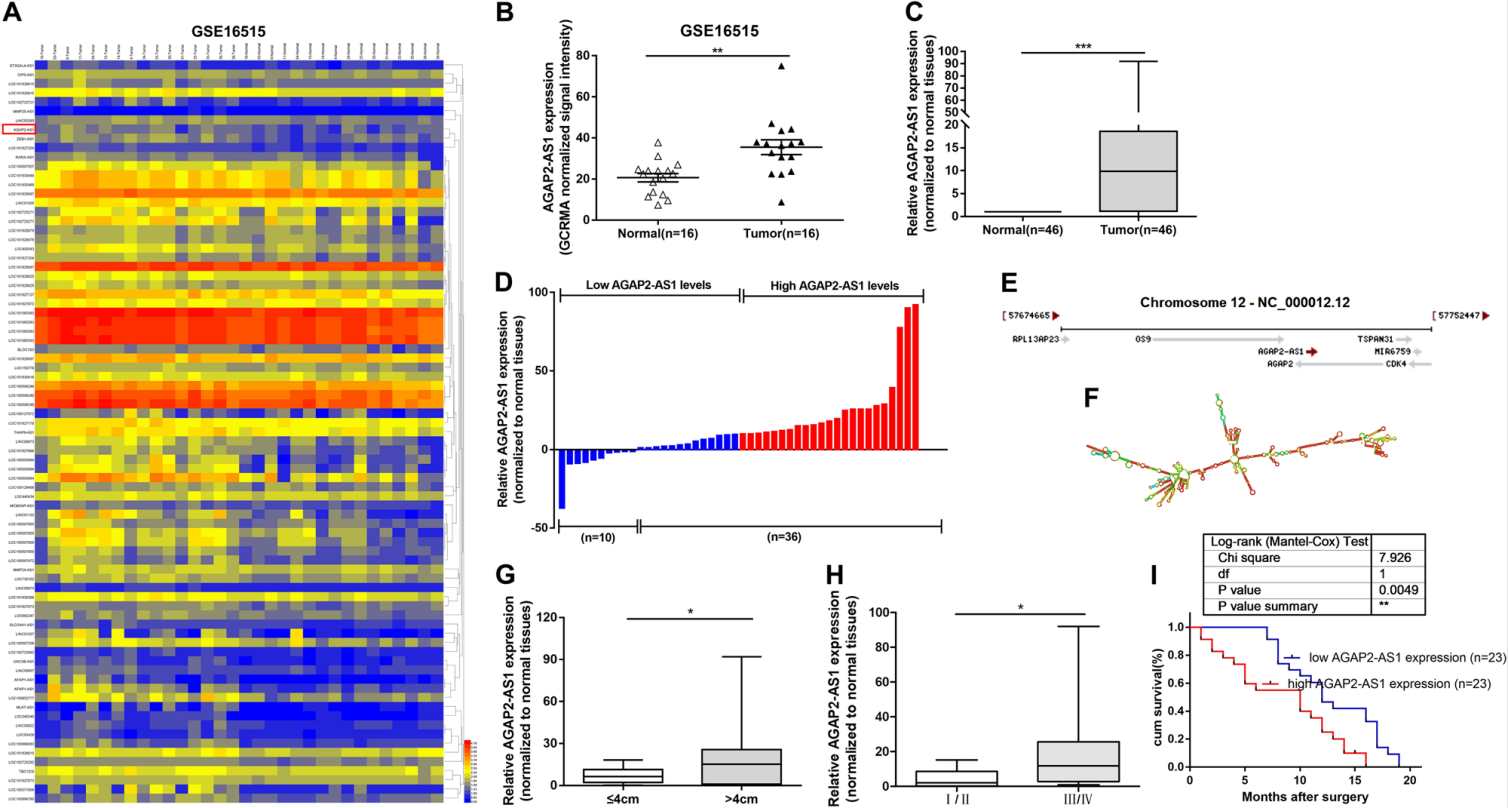
Expression of AGAP2-AS1 in pancreatic cancer (PC) tissues and its clinical parameters. **a** The GSE16515 data set containing lncRNAs profiles of 16 human PC tissues and 16 corresponding para-cancerous tissues was analyzed, and lncRNAs with *P* < 0.05 and logFC > 0 in the original normalized signal data were screened out. The original normalized signal data was log10 converted. **b** AGAP2-AS1 expression of GCRMA normalized signal intensity in PC tissues (*n* = 16) compared with noncancerous tissues (*n* = 16) analyzed using microarray data from Gene Expression Omnibus data sets. **c** Relative expression levels of AGAP2-AS1 in PC tissues (*n* = 46) compared with adjacent non-tumor tissues (*n* = 46). AGAP2-AS1 expression was examined by quantitative real-time PCR (qRT-PCR) and normalized to GAPDH expression. The expression levels of AGAP2-AS1 in tumor tissues relative to normal tissues were calculated as [(^AGAP2−AS1^CT_normal_–^GAPDH^CT_normal_) − (^AGAP2−AS1^CT_tumor_–^GAPDH^CT_tumor_)]. **d** Samples were classified into relatively high- and low-expression group according to the median value of the AGAP2-AS1 expression level in PC tissues. **e** AGAP2-AS1 as published in the NCBI database (NR_027032.1). **f** Prediction of AGAP2-AS1 structure based on minimum free energy (MFE) and partition function. The color scale indicates the confidence for the prediction for each base, with shades of red indicating strong confidence. **g** Patients with larger tumors (>4 cm) had higher levels of AGAP2-AS1 expression than patients with smaller tumors (≤4 cm). **h** Comparison of the expression levels of AGAP2-AS1 in patients with higher pathological stage (III/IV) and those with lower pathological stage (I/II), these were significantly higher in the latter case. **i** Kaplan–Meier overall survival (OS) curves of patients with pancreatic cancer according to AGAP2-AS1 expression levels. *P < 0.05, **P < 0.01, ***P < 0.001

Based on bioinformatics analysis, we downloaded a microarray data set (GSE16515) containing 16 human PC tissues and 16 corresponding para-cancerous tissues from the Gene Expression Omnibus (GEO), and searched for lncRNAs that were significantly differentially expressed in PC Specifically, we selected lncRNAs for which *P* < 0.05 and logFC (fold change) > 0 for the difference between human PC tissues and corresponding para-cancerous tissues in original normalized signal data. Log10 conversion was used to process the original normalized signal data (Fig. 1a). We found that lncRNA AGAP2-AS1 (AGAP2 antisense RNA 1) was upregulated in the GSE16515 data set (Fig. 1b). According to NCBI (Gene, NR_027032.1), AGAP2-AS1 (2117 bp) is a lncRNA with only one transcript (1567 bp). Given that AGAP2-AS1 has a carcinogenic role in other tumor types, including gastric cancer^16,17^ and non–small-cell lung cancer cells, and its function and mechanism in PC have not been reported, we focused on it in particular. To verify the expression results from the microarray, we determined the expression levels of AGAP2-AS1 in 46 pairs of PC and normal tissues by quantitative real-time polymerase chain reaction (qRT-PCR) (Fig. 1c). As shown in Fig. 1d, AGAP2-AS1 expression was upregulated in 78.3% (36 out of 46) PC tissues (13.40306 ± 23.17950). AGAP2-AS1 is located on chromosome 12q14.1 (Fig. 1e) and has a transcript length of 1567 nucleotides. ViennaRNA Web Services (http://rna.tbi.univie.ac.at/) was used to predict the RNA secondary structure of AGAP2-AS1 based on the minimum free energy (Fig. 1f).

Subsequently, we investigated whether there was a correlation between expression levels of AGAP2-AS1 and clinical pathological factors of PC patients. Elevated levels of AGAP2-AS1 were positively correlated with greater tumor size (21.08339 ± 25.68411 vs. 6.92280 ± 5.46706, *P* = 0.0254) (Fig. 1g) and higher tug-lymph node metastasis (TNM) stage (20.81571 ± 24.12158 vs. 4.76958 ± 5.43756, *P* = 0.0137) (Fig. 1h). Then, according to the median AGAP2-AS1 expression level, the samples were divided into a high AGAP2-AS1 expression group (above median value, *N* = 23) and low AGAP2-AS1 expression group (below median value, *N* = 23) (Fig. 1d). We performed a chi-square test to compare the clinicopathological features of the two groups and obtained the data shown in Table 1; the upregulation of AGAP2-AS1 expression in PC tissues was significantly associated with tumor size (*P* = 0.0010), late TNM staging (*P* = 0.0020), and lymph node metastasis (*P* = 0.0020) in PC patients. However, histological grade, gender, and age were found to have no significant correlation with AGAP2-AS1 expression (Table 1). We used Kaplan–Meier analysis to assess the effect of AGAP2-AS1 expression on overall survival (OS) and to determine the relationship between AGAP2-AS1 expression and prognosis. It was concluded that overexpression of AGAP2-AS1 was associated with poor prognosis in PC patients (*P* = 0.0049) (Fig. 1i).

**Table 1:** Correlation between AGAP2-AS1 expression and clinicopathological characteristics of 46 PC patients

Characteristics	Expression of AGAP2-AS1		*P* value*
	Low (*n* = 23)	High (*n* = 23)	Chi-squared test
*Sex*			0.1343
Male	16	11	
Female	7	12	
*Age*			0.1389
≤60	15	10	
>60	8	13	
*Histological grade*			0.0699
Low or undiffer	17	11	
Middle or high	6	12	
*TNM stage*			0.0020**
I and II	13	3	
III and IV	10	20	
*Tumor size*			0.0010**
≤4 cm	15	4	
>4 cm	8	19	
*Regional lymph node invasion*			0.0020**
Negtive	13	3	
Positive	10	20	
*Distant metastasis*			0.0277*
Negtive	19	12	
Positive	4	11	

*P < 0.05, **P < 0.01, ***P < 0.001

### RREB1 induces transcription of AGAP2-AS1

**Fig. 2:**
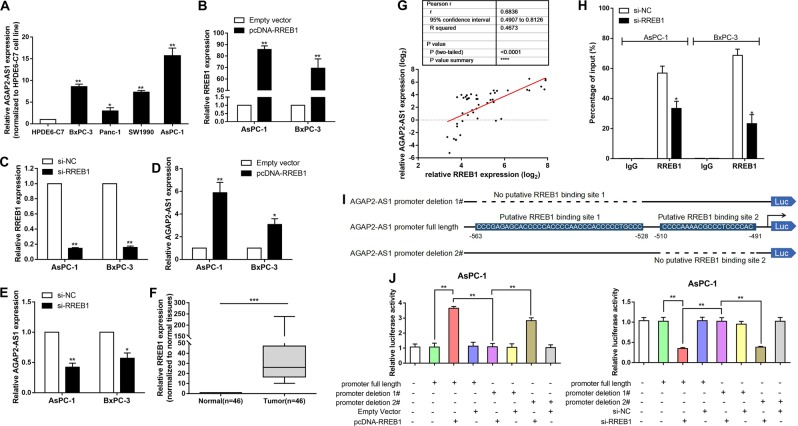
RREB1 induces transcription of AGAP2-AS1. **a** AGAP2-AS1 expression levels in human PC cell lines BxPC-3, SW1990, PANC-1, and AsPC-1 compared with those in human normal pancreatic ductal epithelial cells (HPDE6-C7) as determined by qRT-PCR. **b** Relative expression levels of RREB1 in AsPc-1 and BxPC-3 cells treated with empty vector or pcDNA-RREB1 as measured using qPCR. **c** Relative expression levels of RREB1 in AsPc-1 and BxPC-3 cells transfected with si-NC or si-RREB1 as measured using qPCR. **d** Relative expression levels of AGAP2-AS1 in AsPc-1 and BxPC-3 cells treated with empty vector or pcDNA-RREB1 as measured using qRT-PCR. **e** Relative expression levels of AGAP2-AS1 in AsPc-1 and BxPC-3 cells transfected with si-NC or si-RREB1 as measured using qRT-PCR. **f** Relative expression levels of RREB1 in PC tissues (*n* = 46) compared with para-carcinoma tissues (*n* = 46). **g** Relationship between AGAP2-AS1 expression and RREB1 mRNA levels in PC tissues (*n* = 46) compared with corresponding nontumor tissues (*n* = 46). **h** Enrichment of RREB1 in the AGAP2-AS1 promoter after RREB1 knockdown. After RREB1 was immunoprecipitated, we detected the promoter region containing RREB1-binding sequences by qRT-PCR. The ChIP primers are listed in Supplementary Table S1. **i** Description of RREB1-binding sites in the promoter region of AGAP2-AS1 and the schematic of AGAP2-AS1 promoter deletion 1# and AGAP2-AS1 promoter deletion 2# luciferase reporter vectors. **j** Relative luciferase activities as analyzed in AsPc-1 cells co-transfected with pcDNA-RREB1/empty vector or si-RREB1/si-NC and luciferase reporter vectors (AGAP2-AS1 promoter full length, AGAP2-AS1 promoter deletion 1#, and AGAP2-AS1 promoter deletion 2#). *P < 0.05, **P < 0.01, ***P < 0.001

To explore the biological mechanism underlying high expression of AGAP2-AS1, we examined the expression levels of AGAP2-AS1 in PC cell lines. As shown in Fig. 2a, the expression levels of AGAP2-AS1 in human PC BxPC-3, SW1990, and PANC-1 cells, and in the human metastatic PC cell line AsPC-1, were higher than those in human normal pancreatic ductal epithelial cells (HPDE6-C7). The expression of AGAP2-AS1 was most significantly upregulated in AsPC-1 (*P* < 0.01) and BxPC-3 (*P* < 0.01) (Fig. 2a).

Then, computational screening based on the Jaspar algorithm (http://jaspardev.genereg.net/) was used to show that there were RREB1-binding sites in the AGAP2-AS1 promoter region (Supplementary Table S2). We discussed whether overexpression of AGAP2-AS1 was mediated by RREB1. An RREB1 overexpression plasmid was used to upregulate RREB1 expression, and RREB1 small interfering RNA (siRNA) was used to downregulate RREB1 expression (Fig. 2b, c). A higher level of AGAP2-AS1 was induced in AsPC-1 and BxPC-3 cells transfected with the RREB1 overexpression plasmid (Fig. 2d). We next attempted to investigate whether high expression of AGAP2-AS1 was mediated by endogenous RREB1. We found that siRNA-mediated knockdown of RREB1 resulted in a decrease in expression levels of AGAP2-AS1 (Fig. 2e). We examined the expression of RREB1 in 46 pairs of PC tissues and para-cancerous normal tissues by qRT-PCR, and found that RREB1 was upregulated in PC tissues (Fig. 2f). As shown in Fig. 2g, the expression level of AGAP2-AS1 was positively correlated with the level of RREB1 in PC tissues.

**Fig. 3:**
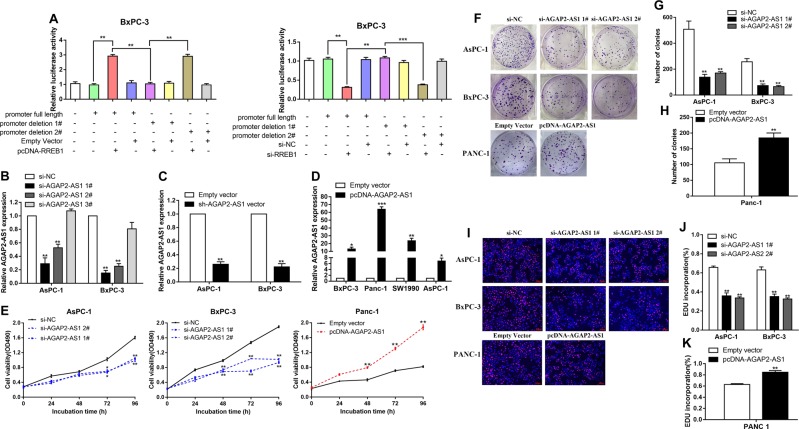
AGAP2-AS1 regulates PC cell proliferation in vitro. **a** Relative luciferase activities were analyzed in BxPC-3 cells co-transfected with pcDNA-RREB1/empty vector or si-RREB1/si-NC and luciferase reporter vectors (AGAP2-AS1 promoter full length, AGAP2-AS1 promoter deletion 1#, and AGAP2-AS1 promoter deletion 2#). **b** Relative expression levels of AGAP2-AS1 in AsPc-1 and BxPC-3 cells transfected with si-NC or si-AGAP2-AS1 (si-AGAP2-AS1 1#, si-AGAP2-AS1 2#, and si-AGAP2-AS1 3#), as measured using qRT-PCR. **c** Relative expression levels of AGAP2-AS1 in AsPc-1 and BxPC-3 cells treated with empty vector and sh-AGAP2-AS1 vector. **d** Relative expression levels of AGAP2-AS1 in PC cell lines treated with empty vector and pcDNA-AGAP2-AS1. **e** MTT assays were carried out to detect the proliferation of AsPc-1 and BxPC-3 cells transfected with si-AGAP2-AS1, and Panc-1 cells treated with pcDNA-AGAP2-AS1. **f**–**h** Colony-forming experiments were performed to detect the viability of AsPc-1 and BxPC-3 cells transfected with si-AGAP2-AS1, and Panc-1 cells treated with pcDNA-AGAP2-AS1. **i**–**k** EdU staining assays were used to determine the proliferation of AsPc-1 and BxPC-3 cells transfected with si-AGAP2-AS1, and Panc-1 cells treated with pcDNA-AGAP2-AS1. *P < 0.05, **P < 0.01, ***P < 0.001

The results of chromatin immunoprecipitation (ChIP) experiments showed that RREB1 could bind to the promoter region of AGAP2-AS1. Upregulation of RREB1 led to occupancy of the AGAP2-AS1 locus (Fig. 2h). To further determine the function of the RREB1-binding sites, we cloned the promoter region of AGAP2-AS1 into a luciferase reporter plasmid and made deletions at the promoter of AGAP2-AS1 (Fig. 2i). AsPc-1 and BxPC-3 cells were co-transfected with the pcDNA-RREB1/empty vector or si-RREB1/scrambled negative control siRNA (si-NC) and luciferase reporter vectors AGAP2-AS1 promoter full length, AGAP2-AS1 promoter deletion 1#, or AGAP2-AS1 promoter deletion 2#. AGAP2-AS1 promoter deletion 1# caused a significant reduction in promoter activity compared with the full-length promoter construct and AGAP2-AS1 promoter deletion 2# (Figs. 2j and 3a). Taken together, these data confirm that AGAP2-AS1 is frequently increased in PC, and suggest that RREB1-induced AGAP2-AS1 promoter activation may partially explain this disorder.

### AGAP2-AS1 regulates PC cell proliferation and apoptosis in vitro

**Fig. 4:**
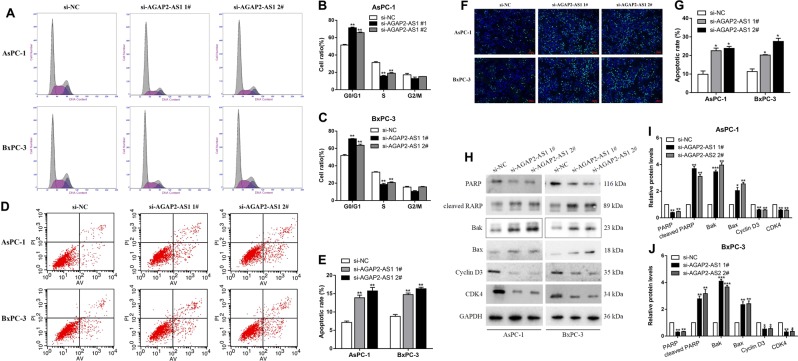
AGAP2-AS1 regulates PC cell cycle arrest and apoptosis in vitro. **a**–**c** Flow cytometry was used to determine and compare the differences in cell cycle progression when PC cells were transfected with si-NC and si-AGAP2-AS1. The bar graph represents percentage data for G0/G1, S, and G2/M phase cells. **d**, **e** Flow cytometry was used to determine and compare apoptosis differences in PC cell lines of the si-NC group and si-AGAP2-AS1 group. LL dead cells, UL viable cells, LR early apoptotic cells, UR terminal apoptosis cells. **f**, **g** A TUNEL staining assay was used to determine apoptosis of AsPc-1 and BxPC-3 cells transfected with si-NC and si-AGAP2-AS1. Green, apoptotic cells; blue, DAPI stained nuclei. **h**–**j** Protein levels of apoptosis-related proteins (PARP, cleaved PARP, Bak, and Bax) and G1–S-phase checkpoint proteins (such as cyclin D3 and CDK4) were detected by western blot analysis in AsPC-1 and BxPC-3 cells transfected with si-NC and si-AGAP2-AS1. *P < 0.05, **P < 0.01, ***P < 0.001

**Fig. 5:**
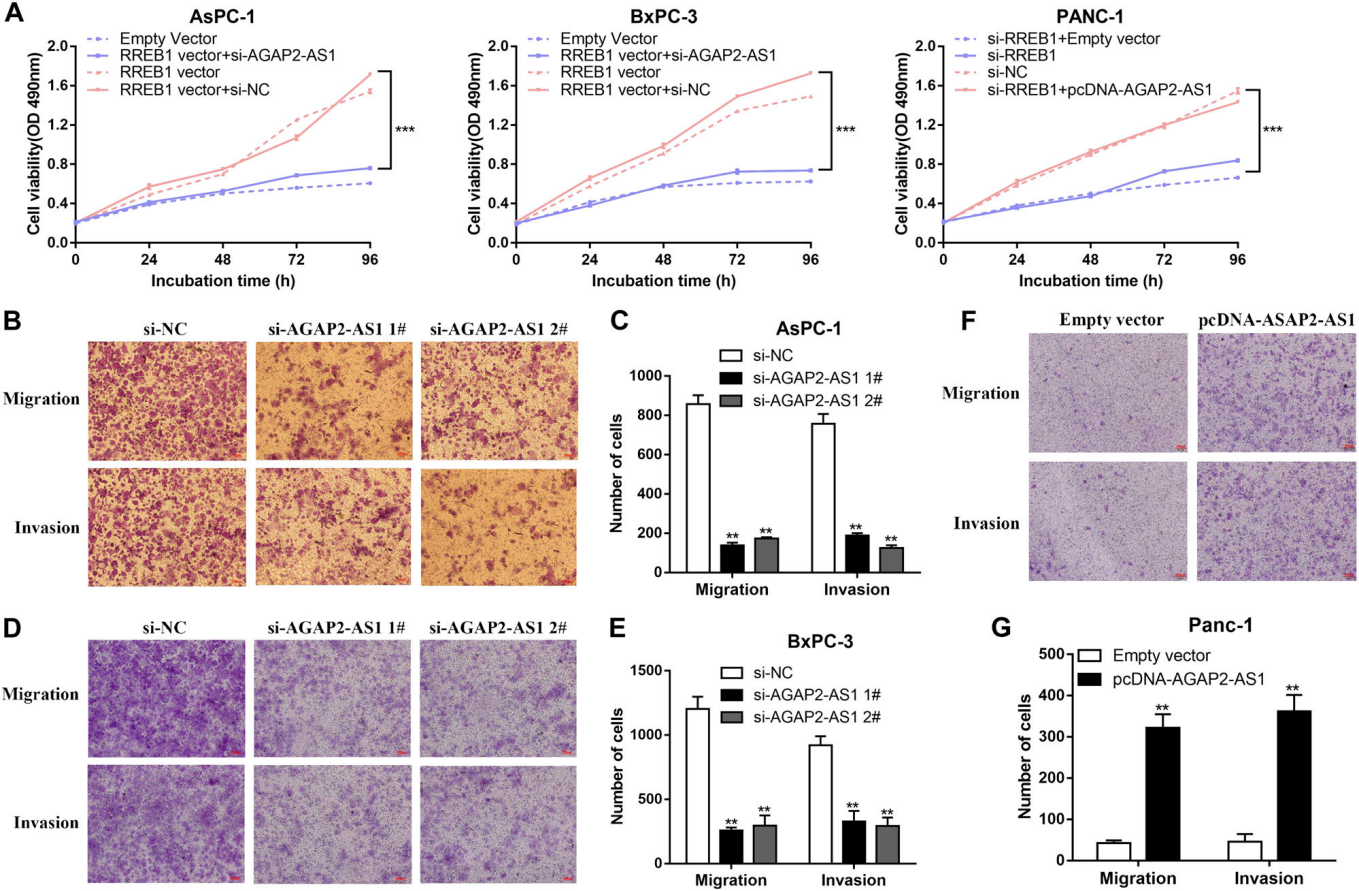
AGAP2-AS1 facilitates PC cell migration and invasion in vitro. MTT assays were performed to detect the proliferation of AsPc-1 and BxPC-3 cells treated with empty vector, pcDNA-RREB1, pcDNA-RREB1 + si-NC, or pcDNA-RREB1 + si-AGAP2-AS1, and of PANC-1 cells treated with si-NC, si-RREB1, si-RREB1 + empty vector, or si-RREB1 + pcDNA-AGAP2-AS1. **b**, **c** Transwell assays were performed with si-AGAP2-AS1-transfected AsPC-1 cells to determine the effects of AGAP2-AS1 on cell migration and invasion. **d**, **e** Transwell assays were performed with si-AGAP2-AS1-transfected BxPC-3 cells to determine the effects of AGAP2-AS1 on cell migration and invasion. **f**, **g** Transwell assays were performed with pcDNA-AGAP2-treated PANC-1 cells to determine the effects of AGAP2-AS1 on cell migration and invasion. The cells on the underside of the membrane were stained and counted. *P < 0.05, **P < 0.01, ***P < 0.001

To evaluate the effects of AGAP2-AS1 on cellular processes, we designed siRNAs and short hairpin RNAs (shRNAs) to silence AGAP2-AS1 expression in AsPC-1 and BxPC-3 cells (Fig. 3b, c). Plasmid-mediated overexpression was used for exogenous manipulation of AGAP2-AS1 expression in PC cell lines; we selected the PANC-1 cell line for overexpression experiments, because pcDNA-AGAP2-AS1 showed the highest overexpression efficiency in PANC-1 (Fig. 3d). MTT analysis showed that knockdown of AGAP2-AS1 expression could inhibit cell proliferation compared with a control group (Fig. 3e). This conclusion was also confirmed by a colony formation assay and EdU assay. By contrast, overexpression of AGAP2-AS1 promoted cell proliferation (Fig. 3f–k). Next, we performed flow cytometry to examine whether AGAP2-AS1 could affect the proliferation of PC cells by altering cell cycle progression. The results showed that the cell cycle progression of si-AGAP2-AS1 cells was arrested in the G1–G0 phase and the percentage of S-phase cells was decreased compared with the cells transfected with a si-NC (Fig. 4a–c). There was also an increase in the fraction of apoptotic cells following siRNA treatment, compared with controls (Fig. 4d, e). This conclusion was confirmed by the terminal deoxynucleotidyl transferase-mediated dUTP nick-end labeling (TUNEL) assay (Fig. 4f, g). Moreover, si-AGAP2-AS1 cells expressed significantly higher levels of apoptosis-related proteins, including cleaved PARP, Bak, and Bax. Consistent with the cell cycle progression data, the expression levels of G1–S-phase checkpoint proteins (such as cyclin D3 and CDK4) were markedly decreased when AGAP2-AS1 was silenced (Fig. 4h–j). These data indicate that inhibition of PC cell proliferation by AGAP2-AS1 silencing could be attributed to enhanced apoptosis and cell cycle arrest at the G1–S checkpoint. In addition, in order to further verify the relationship between RREB1 and AGAP2-AS1, rescue experiments were conducted. Co-transfection with the RREB1 plasmid and si-AGAP2-AS1 partially abolished the RREB1-activated acceleration in cell growth, while co-transfection with the si-RREB1 and AGAP2-AS1 plasmid partially reversed the inhibition of cell growth induced by si-RREB1 (Fig. 5a).

### AGAP2-AS1 facilitates PC cell migration and invasion in vitro

Tumor cell migration and invasion are important aspects of cancer progression. Here, we carried out a transwell assay to investigate the effects of AGAP2-AS1 on PC cell migration and invasion. Compared with the control group, knockdown of AGAP2-AS1 expression blocked the migration and invasion of AsPC-1 and BxPC-3 cells (Fig. 5b–e), while upregulation of AGAP2-AS1 promoted the migration and invasion of PANC-1 cells (Fig. 5f, g). In summary, these data indicate that AGAP2-AS1 has an important role in facilitating PC cell migration and invasion.

### Knockdown of AGAP2-AS1 inhibits PC proliferation in vivo

**Fig. 6:**
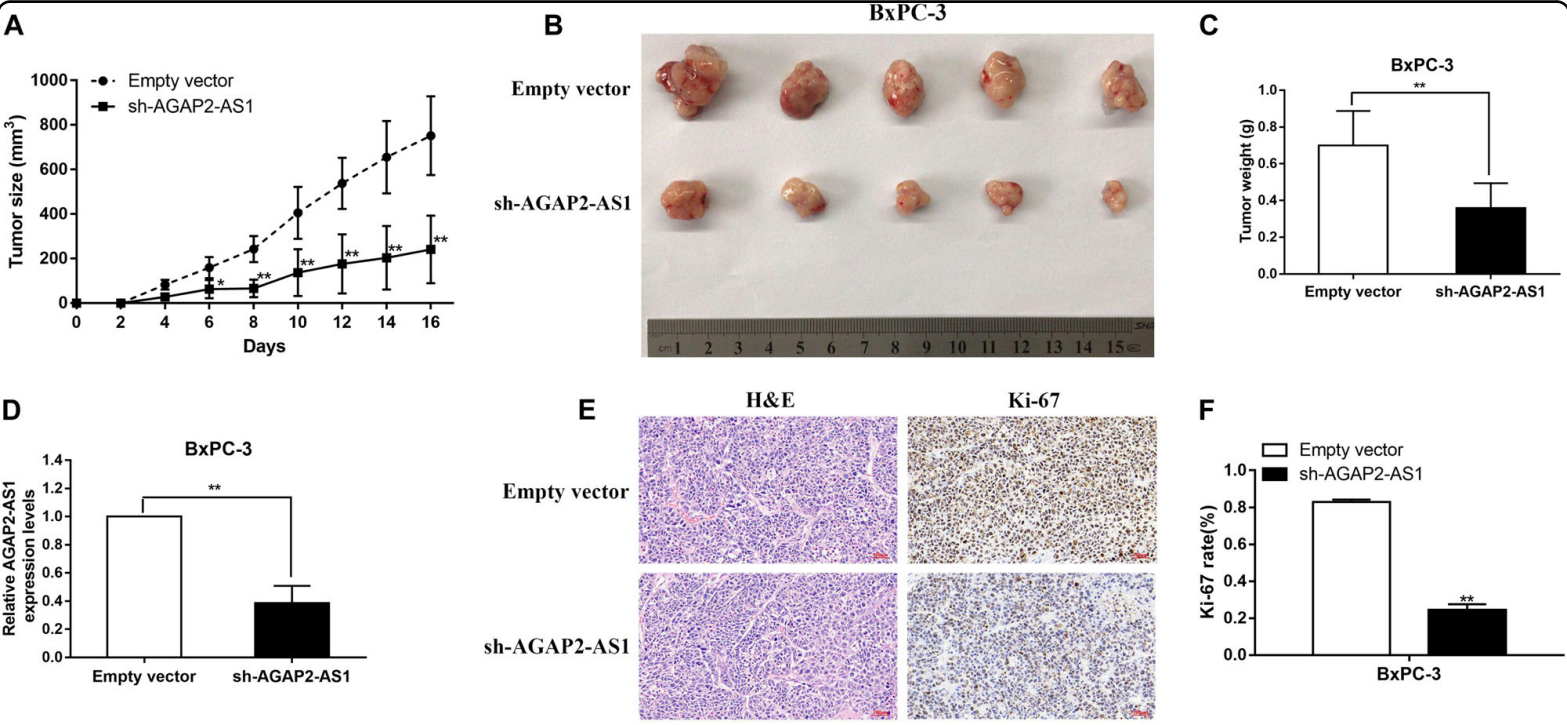
AGAP2-AS1 promotes the tumorigenesis of PC cells in vivo. Tumor volumes were measured and recorded every 2 days after injection. The mean tumor volume is represented by dots and the bars represent SD (*n* = 5). **b** BxPC-3 cells transfected with empty vectors and sh-AGAP2-AS1 were injected into nude mice (*n* = 5) with the same concentration and amount. **c** Tumors were removed and weighed and recorded on day 16 postinjection; their weights are expressed as mean tumor weight ± SD. **d** The expression levels of AGAP2-AS1 in xenograft tumors were detected by qRT-PCR (*n* = 5). **e**, **f** Tumor sections were examined using H&E and IHC staining with antibodies against Ki-67. Error bars represent mean ± standard error. *P < 0.05, **P < 0.01, ***P < 0.001

To further explore the biological function of AGAP2-AS1 in PC in vivo, we constructed a xenograft tumor model in nude mice using the human PC cell line BXPC-3 transfected with an empty vector or sh-AGAP2-AS1. Sixteen days after subcutaneous injection of BxPC-3 into nude mice, the sh-AGAP2-AS1 group showed a markedly lower speed of tumor growth compared with the empty vector group (Fig. 6A), as well as reduced tumor volume and weight (Fig. 6b, c), indicating that AGAP2-AS1 regulates PC cell proliferation in vivo. As shown in Fig. 6d, qRT-PCR confirmed that there was a lower level of AGAP2-AS1 expression in tumor tissues from sh-AGAP2-AS1-transfected cells. Furthermore, upon immunohistochemistry (IHC) analysis, tumors grown from BxPC-3/sh-AGAP2-AS1 cells showed weaker staining of Ki-67 than those grown from control cells (Fig. 6e, f).

### AGAP2-AS1 promotes PC proliferation and migration by inhibiting the expression of ANKRD1 and ANGPTL4

**Fig. 7:**
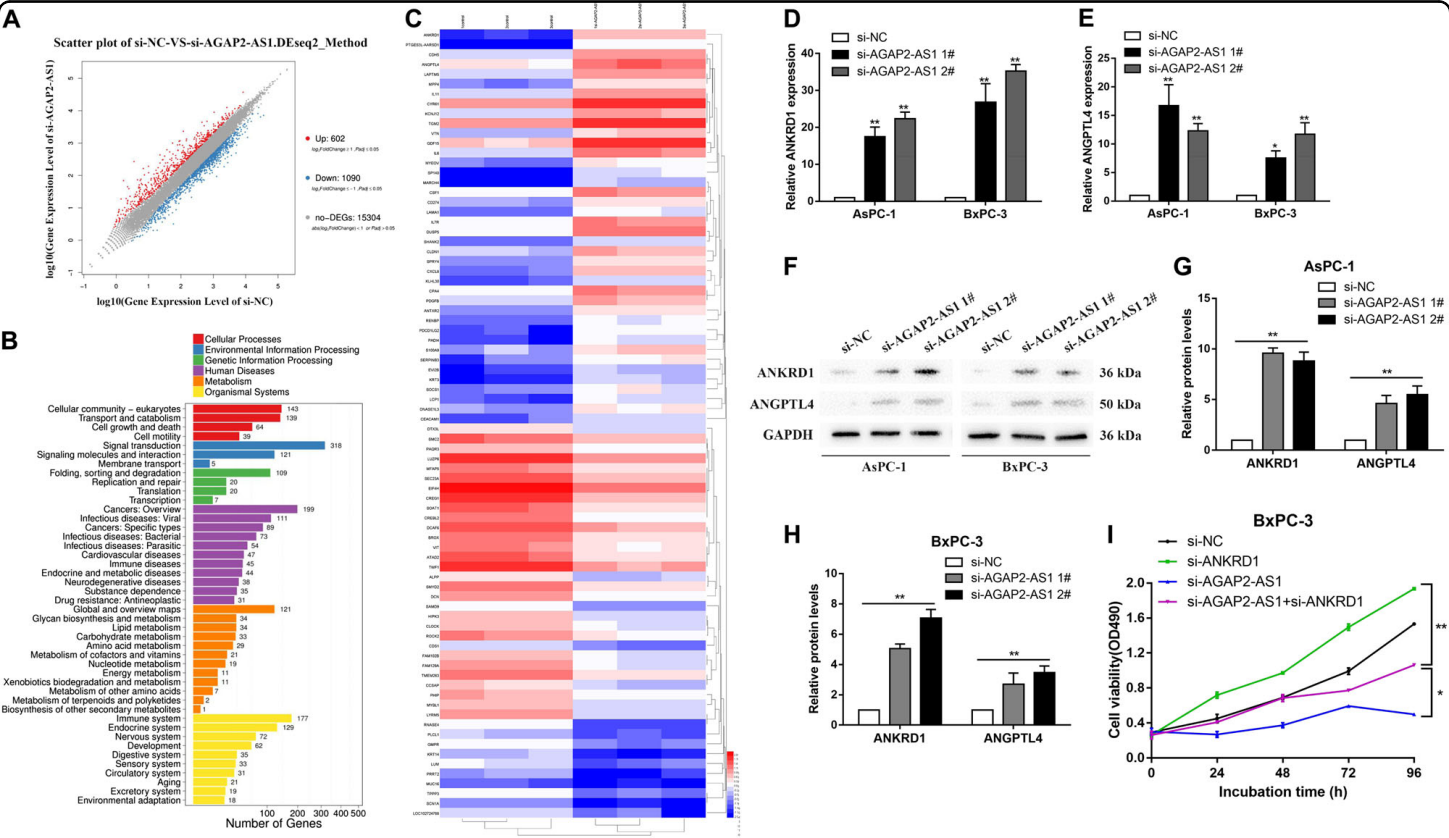
AGAP2-AS1 inhibits the expression of ANKRD1 and ANGPTL4 in PC cells. **a** A set of 602 mRNAs showed an increase in abundance of log_2_FC ≥ 1, while AGAP2-AS1 also reduced the abundance of 1090 genes (log_2_FC ≤ −1) in RNA profiles, based on RNA transcriptome sequencing of the control group and the siRNA-AGAP2-AS1 group. **b** GO analysis showed that the influenced genes could be grouped into six categories on the basis of the KEGG pathway involved: cellular processes, environmental information processing, genetic information processing, human diseases, metabolism, and organismal systems. **c** Heat map showing the expression change of the most variable 40 genes in BxPC-3 cells after transfection with si-NC and si-AGAP2-AS1. Gene expression is shown as RPKM after log10 conversion. **d**, **e** Relative expression levels of ANKRD1 and ANGPTL4 in AsPc-1 and BxPC-3 cells transfected with si-NC or si-AGAP2-AS1 were measured using qRT-PCR. **f**–**h** ANKRD1 and ANGPTL4 protein levels were detected by western blot analysis in AsPC-1 and BxPC-3 cells transfected with si-NC and si-AGAP2-AS1. **i** MTT experiments were performed to determine the cell viability of BxPC-3 cells transfected with si-NC, si-ANKRD1, si-AGAP2-AS1, si-AGAP2-AS1 + si-ANKRD1. *P < 0.05, **P < 0.01, ***P < 0.001

**Fig. 8:**
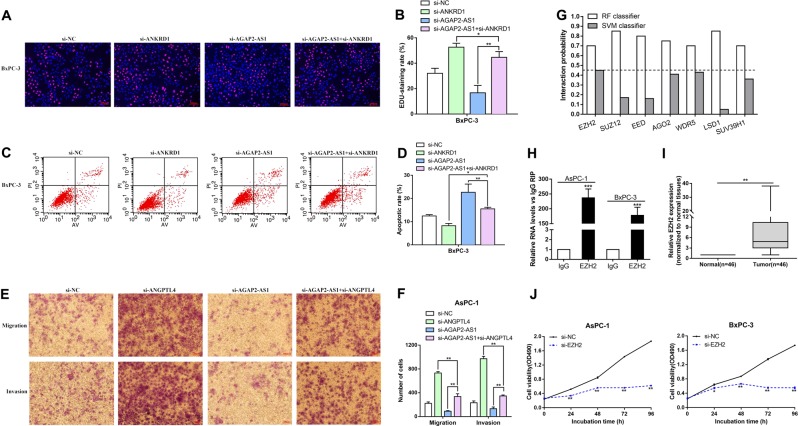
Co-transfection partially reversed the apoptosis acceleration, proliferation inhibition, and migration inhibition induced by si-AGAP2-AS1. **a**, **b** EdU assays were performed to determine the cell viability for BxPC-3 cells transfected with si-NC, si-ANKRD1, si-AGAP2-AS1, si-AGAP2-AS1 + si-ANKRD1. **c**, **d** Flow cytometry assays were performed to analyze the cell apoptosis in for BxPC-3 cells transfected with si-NC, si-ANKRD1, si-AGAP2-AS1, and si-AGAP2-AS1 + si-ANKRD1. **e**, **f** Transwell assays were used to determine cell migration and invasion in AsPc-1 and BxPC-3 cells transfected with si-NC, si-ANGPTL4, si-AGAP2-AS1, and si-AGAP2-AS1 + si-ANGPTL4. **g** Bioinformatics prediction of the interaction probability of AGAP2-AS1 and RNA-binding proteins on a random forest (RF) or support vector machine (SVM) basis. **h** In the RIP experiments, co-precipitated RNA was detected by qRT-PCR. The fold enrichment of AGAP2-AS1 in the AsPc-1 and BxPC-3 cell was matched using IgG as a control. **i** Relative expression level of EZH2 in PC tissue (*n* = 46) compared with adjacent nontumor tissue (*n* = 46). Standardization was performed with reference to GAPDH expression. **j** MTT assays were performed to determine the cell viability for AsPc-1 and BxPC-3 cells transfected with si-EZH2. *P < 0.05, **P < 0.01, ***P < 0.001

To probe the AGAP2-AS1-related pathway in PC without bias, we evaluated the gene expression profiles of BxPC-3 cells with knockdown of AGAP2-AS1 by RNA transcriptome sequencing of a control group and the siRNA-AGAP2-AS1 group. As shown in Fig. 7a, a set of 602 mRNAs showed an increase in abundance of log_2_FC ≥ 1, while AGAP2-AS1 also reduced the abundance of 1090 genes (log_2_FC ≤ −1). GO analysis showed that the most significantly influenced biological processes were pathways involving cellular community, transport and catabolism, cell growth and death, and cell motility (Fig. 7b). The most variable 40 mRNAs in terms of upregulation and downregulation are shown in Fig. 7c. The most strongly upregulated mRNA, ankyrin repeat protein 1 (ANKRD1), is a tumor suppressor gene that positively regulates apoptosis^18,19^. Another significantly upregulated mRNA, angiopoietin-like 4 (ANGPTL4), has been reported to prevent metastasis by inhibiting blood vessel growth and tumor cell motility and invasiveness^18–20^. After knockdown of AGAP2-AS1 in AsPC-1 and BxPC-3 cells, expression levels of ANKRD1 and ANGPTL4 were detected by qRT-PCR, and the results were consistent with those of the sequencing (Fig. 7d, e). In addition, western blot analysis confirmed that ANKRDl and ANGPTL4 were upregulated at the protein level after knockdown of AGAP2-AS1 (Fig. 7f–h). To investigate whether AGAP2-AS1 regulated PC cell apoptosis and proliferation by inhibiting ANKRD1 expression, BxPC-3 cells were co-transfected with si-AGAP2-AS1 and si-ANKRD1. MTT assay (Fig. 7i), EdU assay (Fig. 8a, b), and flow cytometry (Fig. 8c, d) showed that co-transfection partially rescued the acceleration of apoptosis and inhibition of proliferation induced by si-AGAP2-AS1. At the same time, in order to investigate whether AGAP2-AS1 reduced the invasion and metastasis of PC cells by inhibiting ANGPTL4 expression, AsPC-1 cells were co-transfected with si-AGAP2-AS1 and si-ANGPTL4. Results of a transwell assay showed that co-transfection could partially rescue the inhibition of invasion and migration induced by si-AGAP2-AS1 in AsPC-1 cells (Fig. 8e, f).

### AGAP2-AS1 downregulates ANKRD1 and ANGPTL4 by interacting with EZH2

An increasing number of studies have shown that lncRNAs promote epigenetic activation or gene expression silencing by binding to specific RNA-binding proteins (RBPs)^21^. Thus, we performed a bioinformatics analysis to predict RBPs that might interact with AGAP2-AS1 (http://pridb.gdcb.iastate.edu/RPISeq/). This showed that AGAP2-AS1 might bind to EZH2, SUZ12, EED, AGO2, WDR5, LSD1, and SUV39H1 (Fig. 8g). The methyltransferase PRC2 is composed of EZH2, SUZ12, and EED, and catalyzes the dimethylation and trimethylation of lysine residue 27 of histone 3 (H3K27me3) to epigenetically inhibit expression of certain genes^22^.

**Fig. 9:**
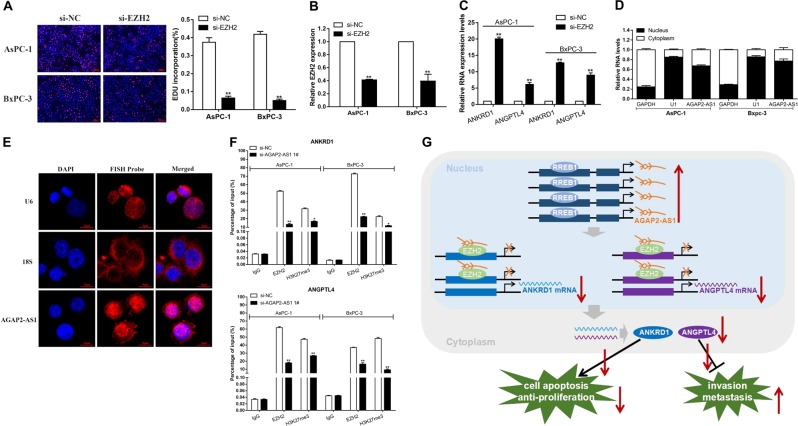
AGAP2-AS1 downregulates ANKRD1 and ANGPTL4 by interacting with EZH2. **a** EdU assays were performed to determine the cell proliferation in AsPc-1 and BxPC-3 cells transfected with si-EZH2. **b** The relative expression levels of EZH2 in AsPc-1 and BxPC-3 cells transfected with si-NC or si-EZH2 were measured using qPCR. **c** The relative expression levels of ANKRD1 and ANGPTL4 in AsPc-1 and BxPC-3 cells transfected with si-NC or si-EZH2 were measured using qRT-PCR. **d** The cytoplasm and nuclei of AsPC-1 and BxPC-3 cells were isolated, and the levels of AGAP2-AS1 were detected by qRT-PCR with GAPDH as the cytoplasmic control, U1 as the nuclear control. The distribution of AGAP2-AS1 is expressed as a percentage of total RNA. **e** Confocal FISH images showing localization of AGAP2-AS1 in BxPC-3 cells. U6 was taken as representative of nuclear localization, and 18s as representative of cytoplasmic localization. Red, FISH probe; blue, DAPI nuclear staining. **f** ChIP results showing EZH2 occupancy on the ANKRD1 and ANGPTL4 promoter regions; knockdown of AGAP2-AS1 decreases their occupancy. IgG was used as a negative control. **g** Schematic diagram of the mechanism of AGAP2-AS1/EZH2/ANKRD1 and ANGPTL4 axis in PC cells. *P < 0.05, **P < 0.01, ***P < 0.001

In previous studies, about 20% of human lncRNAs have been verified to interact with PRC2, and it has been proposed that some lncRNAs may have the ability to recruit comb proteins to their target genes^23^. In addition, PRC2 aberrations are closely related to carcinogenesis^24^. We further performed RNA-binding protein immunoprecipitation (RIP) analysis, confirming that AGAP2-AS1 could interact with EZH2 (Fig. 8h). To investigate the role of EZH2 in PC, we performed qRT-PCR analysis and found a significant increase in EZH2 expression levels in 46 pairs of PC tissues (Fig. 8i). Further experimental analysis showed that knockdown of EZH2 also inhibited PC cell proliferation, similar to the effect of silencing AGAP2-AS1 (Figs. 8j and 9a). In addition, EZH2 siRNA was used to transfect AsPC-1 and BxPC-3 cells (Fig. 9b). The results showed that EZH2 expression was efficiently knocked down, while ANKRD1 and ANGPTL4 mRNA levels were increased (Fig. 9c). Subcellular division localization and fluorescence in situ hybridization assays (FISH) assays were carried out to demonstrate the localization of AGAP2-AS1 in the nuclei and cytoplasm of PC cells; as shown in Fig. 9d, e, AGAP2-AS1 was mainly distributed in the nucleus. Therefore, we hypothesized that AGAP2-AS1 might inhibit the expression of target genes at the transcriptional level by recruiting PRC2. In order to determine whether AGAP2-AS1 participated in transcriptional repression by recruiting PRC2 to the target gene promoter, we performed ChIP analysis by AGAP2-AS1 knockdown. The ChIP assay showed that AGAP2-AS1 reduced the binding of EZH2 and H3K27me3 levels of the ANKRD1 and ANGPTL4 promoters (Fig. 9f). Taken together, our results illustrate that the knockdown of AGAP2-AS1 can inhibit the proliferation, invasion, and migration of PC cells and suppressed the progression of PC through the AGAP2-AS1/EZH2/ANKRD1 and ANGPTL4 axis (Fig. 9g).

## Discussion

There have been increasing numbers of reports of lncRNAs as novel molecular star involved in cell development and human diseases, particularly cancer^14^. LncRNAs are involved in the progression of cancer-regulated gene expression through various mechanisms, including chromatin modification, genomic imprinting, RNA decay, and sponge-like miRNAs^25,26^. In our current study, we used a separate microarray data set (GSE16515) from GEO to perform a comprehensive analysis of aberrantly expressed lncRNAs in PC. Thus, we showed that expression of PC-associated lncRNA AGAP2-AS1 was significantly higher in PC tissues than in corresponding nontumor tissues. The upregulation of AGAP2-AS1 was positively correlated with tumor invasion depth and TNM stage of PC. In addition, the abnormal upregulation of AGAP2-AS1 in PC tissues was related to poor prognosis. This lncRNA may have potential as a new and better prognostic indicator.

Based on the above results, we reasonably proposed that AGAP2-AS1 might have an important role in the progression of PC. We found that upregulation of AGAP2-AS1 also promoted apoptosis arrest and PC cell proliferation in vivo. These findings indicated that AGAP2-AS1 exerts a carcinogenic effect in PC, and that its overexpression promotes the occurrence and development of PC tumors. In addition to our study, Li et al.^27^ reported that upregulated AGAP2-AS1 could downregulate the expression levels of LATS2 and KLF2 by recruiting EZH2 and LSD1 in NSCLS. Another study by Qi et al. found that the upregulation of SP1 by AGAP2-AS1 regulated the development and progression of gastric cancer cells by inhibiting the expression of P21 and E-cadherin^28^. Our finding, along with the previous studies concerning the important roles of AGAP2-AS2 in non–small-cell lung cancer and gastric cancer suggested that AGAP2-AS2 could serve as a new biomarker with early diagnostic and therapeutic value.

Furthermore, our results showed that AGAP2-AS1 was a direct target of RREB1, and that RREB1-activated transcription of AGAP2-AS1 in PC cell lines. In a previous study, Franklin et al.^29^ found that downregulation of RREB1/ZIP3/zinc during tumorigenesis prevented the aggregation of cytotoxic zinc in malignant cells in PC. In addition, Kent et al.^30^ showed that RREB1 was overexpressed in colorectal tumor tissues and cells, and that the expression of the miR-143/145 primary transcript was negatively correlated with RREB1 expression. Siegfried et al. found that the RRRB1-MKL2 chimeric transcription factor increased expression of MKL2, which regulates neural and myogenic differentiation and can mimic the key role of PAX3 in SNS tumorigenesis. Notable, SNS is a tumor type that is prone to local invasion. In most cases, recurrent gene fusion of the PAX3 gene has emerged^31^. Besides, overexpression of RREB1 has been observed in various types of tumors^30,31^, including PC^29^. In this study, our results indicated that the overexpression of RREB1 contributed to the upregulation of AGAP2-AS1 in PC, and that co-transfection (RREB1 expression plasmid and si-AGAP2-AS1) could partially eliminate the RREB1-induced acceleration in cell proliferation.

Previous studies have shown that many lncRNAs can cooperate with chromatin-modifying enzymes to activate or silence downstream target genes^32,33^. *EZH2*, a subunit of PRC2, catalyzes the dimethylation and trimethylation of H3K27me3 to reduce the expression level of the target gene at the transcriptional level^34,35^. Our study revealed that AGAP2-AS1 played a part in promoting PC by interacting with *EZH2* and catalyzing H3K27me3 in the *ANKRD1* and *ANGPTL4* promoter regions in the nucleus, thereby inactivating the tumor suppressors *ANKRD1* and *ANGPTL4*. Abnormal overexpression of *EZH2* has been found in various tumor types^35–37^. It was also verified that knockdown of *EZH2* expression suppressed cell proliferation in PC cell lines.

*ANKRD1* is a member of the ankyrin repeat protein family [NCBI, Gene, NG_023227.1], and has been reported to be a tumor suppressor gene that positively regulates apoptosis^38,39^. Lei et al. demonstrated that downregulation of *ANKRD1* made ovarian cancer cells sensitive to apoptosis induced by cisplatin and ER stress, which is related to the guidance of *GADD153*. *ANKRD1* has an important role in regulating the apoptosis of ovarian cancer cell lines, and it could represent a new molecular target to increase the sensitivity of ovarian cancer to chemotherapy^40^. Jimenez et al. demonstrated that *ANKRD1*, a YAP1 target gene induced by RASSF1A, was epigenetically silenced in a variety of human cancers. *ANKRD1* could also downregulate TP53, BAX, and *CDKN1A* to reduce colony formation of cancer cells, as well as interacting with p53 to participate in reducing the stability of MDM2; the tumor suppressor effect of *ANKRD1* depended on the presence of p53^41^. In this study, we found that co-transfection with si-AGAP2-AS1 and si-ANKRD1 partially prevented si-AGAP2-AS1 from inducing apoptosis and inhibiting proliferation in the BxPC-3 cell line.

ANGPTL4 encodes a glycosylated, secreted protein containing a C-terminal fibrinogen domain [NCBI, Gene, NG_012169.1]. The encoded protein promotes apoptosis of vascular endothelial cells and reduces tumor metastasis by inhibiting angiogenesis and tumor cell invasion^42^. Zhu et al. demonstrated that ANGPTL4 was able to participate in integrin-dependent survival signaling by activating NADPH oxidase Nox1, thus simulating anchorage conditions and bypassing anoikis by controlling reactive oxygen species^43^. Hsieh et al. showed that expression of ANGPTL4 was inhibited at the transcriptional level in UC cell lines and primary tumor samples compared with adjacent normal bladder epithelial cells. Cell function experiments further demonstrated that high expression of ANGPTL4 effectively inhibited UC cell proliferation, invasion, and migration, and also restrained the xenograft formation in vivo^44^.

In conclusion, AGAP2-AS1 promotes PC cell growth and migration by epigenetically regulating the transcription of ANKRD1 and ANGPTL4 in the nucleus. From a broader perspective, our findings identified AGAP2-AS1 as an important prognostic factor for PC patients, further explored the pathogenesis of PC, and highlighted the importance of lncRNA-guided diagnosis and treatment of PC. However, the underlying mechanism by which AGAP2-AS1 might affect other genes and regulatory pathways was not investigated in this study. This requires further study. Our data suggest that AGAP2-AS1 could be of interest in developing biomarkers and therapeutic targets for PC patients.

## Materials and methods

### LncRNA-expression profile analysis

This study analyzed a PC gene expression data set (GSE16515) extracted from GEO. BAM files and standardized probe-level intensity files were downloaded from the GEO database. We compared the RNA-normalized probe-level intensities of 16 human PC tissues and 16 corresponding para-carcinoma tissues and then screened out differentially expressed lncRNAs between the two groups (*P* < 0.05, logFC > 0). The original normalized signal data after log10-transformation were processed using the HEML software to produce a heat map.

### Sample collection of PC tissues

Forty-six PC tissues and matched adjacent normal tissues were collected from PC patients undergoing surgical resection or percutaneous biopsy at the Second Affiliated Hospital of Nanjing Medical University from 2012 to 2017. None of the participants had undergone radiotherapy or chemotherapy before surgery. Histopathological diagnosis was confirmed by experienced pathologists. All tissue samples were frozen immediately after excision, in tubes containing the RNA-later preservative, and stored in liquid nitrogen until total RNA was extracted. All patients provided written informed consent, and the study protocol was approved by the Ethics committee of Nanjing Medical University (Nanjing, Jiangsu, China) and conducted in accordance with the “Declaration of Helsinki Principles”.

### RNA extraction and qRT-PCR

We used TRIzol reagent (Invitrogen, Grand Island, NY, USA) to extract RNA from PC tumor tissues or PC cell lines according to the manufacturer’s protocol. A 20 µl cDNA was reverse-transcribed from 1 µg total RNA using PrimerScript RT Master Mix (Takara, Dalian, China; Cat. No. RR036A) according to the manufacturer’s instructions. qRT-PCR and data collection were performed on an Applied Biosystems 7500 instrument with SYBR Green (Takara, Dalian, China). The primer sequences used for PCR amplification are given in Supplementary Table S1. The results were normalized to the expression of glyceraldehyde-3-phosphate dehydrogenase (GAPDH). The relative expression of BLACAT1 was calculated using the 2^−^^ΔΔCT^ method, with GAPDH expression as a standard.

### Cell culture

A human normal pancreatic ductal epithelial cell line (HPDE6-C7) and four PC cell lines (AsPC-1, SW1990, PANC-1, and BxPC-3) were obtained from the Institute of Biochemistry and Cell Biology, Chinese Academy of Sciences (Shanghai, China). PC cell lines were cultured in DMEM (Invitrogen) containing 10% fetal bovine serum (FBS), 100 U/ml penicillin and 100 mg/ml streptomycin (Invitrogen, Shanghai, China). All cell lines were cultured at 37 °C and 5% CO_2_ in humidified air.

### Transfection of cell lines

A DNA Midiprep kit (Qiagen, Hilden, Germany) was used to manufacture the plasmid vectors (pcDNA-AGAP2-AS1, sh-AGAP2-AS1, and empty vector) for transfection. To prevent off-target effects, three separate siRNAs and an si-NC were designed for different sites and purchased from Invitrogen. According to the instructions in the kit, we used Lipofectamine 3000 (Invitrogen) to transfect siRNA and plasmids into PC cell lines. All the transfected cells were collected for analysis 48 h after transfection. Primer sequences and siRNA/shRNA sequences are given in Supplementary Table S1.

### Cell proliferation analysis

Cell viability was tested using an MTT kit (Sigma) in accordance with the manufacturer’s instructions, allowing the transfected cells to grow in 96-well plates. We recorded cell proliferation every 24 h after transfection of cells according to the manufacturer’s instructions. All experiments were repeated four times. In the colony formation assay, we distributed a defined number of transfected cells in each well of a six-well plate and continued to culture for 2 weeks in a suitable medium containing 10% FBS. The medium was changed every 3 days during this period. Two weeks later, we fixed the colonies with methanol for 20 min and stained with a phosphate-buffered saline (PBS) solution containing 0.1% crystal violet (Sigma) for 15 min. When performing the EdU incorporation assay, we cultured a certain number of cells in 24-well plates. Then, according to the manufacturer’s instructions, we added 10 μM EdU to each well, and the cells were incubated in an incubator for 2 h. After the incubation, we fixed the cells with 4% formaldehyde for 30 min. After washing, we used the Click-iTR EdU kit to detect EdU, with a detection time of about 30 min. We then stained the cells with DAPI for 10 min and observed cells using a fluorescence microscope (Olympus). We used the Image-Pro Plus 6.0 software (Media Cybernetics) to calculate the EdU incorporation rate. The value was expressed as the ratio of EdU-positive cells to total DAPI-positive cells (blue cells).

### Flow cytometry

Cell cycle and apoptosis were analyzed by flow cytometry, and the transfected cells were harvested by trypsin digestion. An FITC-Annexin V Apoptosis Detection Kit was purchased from BD Biosciences. FITC-Annexin V and propidium iodide were used for double staining in accordance with the manufacturer’s instructions, followed by flow cytometry (FACScan; BD Biosicences, Franklin Lakes, NJ, USA). We first distinguished living cells, dead cells, early apoptotic cells, and apoptotic cells among the AsPC-1 and BxPC-3 cells. The relative proportion of early apoptotic cells in the transfection group and the control group was the target of our comparison. When analyzing the cell cycle, we calculated and compared the percentages of G0–G1, S, and G2–M phase cells in the transfected and control groups by FACScan analysis using a CycleTEST PLUS DNA kit (BD Biosciences) according to the instructions provided. All samples were assayed in triplicate.

### TUNEL staining

In accordance with the manufacturer’s instructions, TUNEL assays were performed on AsPC-1 and BxPC-3 cells using an Apoptosis Detection Kit (Ribobio, China). TUNEL-positive cells were evaluated in a randomly selected field of view with no significant necrosis. The TUNEL index was calculated based on the total number of nuclei and cells with green nucleus. All samples were assayed in triplicate.

### Cell migration analysis

Totally, 5 × 10^4^ transfected PC cells were placed in the upper chamber of the insert (pore size 8 μm; Millipore, Billerica, MA, USA) and cultured in serum-free medium. We added medium containing 10% FBS to the lower dish. After 24 h, the AsPC-1 and BxPC-3 cells remaining on the upper membrane were removed with cotton wool. We treated cells that migrated through the membrane with methanol and 0.1% crystal violet, and observed and counted cells of the transfected and control groups using an inverted microscope (Olympus, Tokyo, Japan). The experiment was repeated three times independently.

### Xenotransplantation mouse model

We purchased five 4-week-old BALB/C male nude mice from the Animal Center of Nanjing University (Nanjing, China), and them under pathogen-free conditions in a laminar flow cabinet. For the in vivo cell proliferation assays, we stably transfected the BxPC-3 cell line with shRNA and an empty vector. After the cells were collected, both groups were resuspended at a density of 2 × 10^7^ cells/ml. Then, 100 μl of the shRNA-transfected cells and 100 μl of the empty vector cells were transplanted subcutaneously to either side of each mouse. We examined the growth of xenograft tumors every 2 days and calculated the tumor volume as length × width^2^ × 0.5. Sixteen days after the injection, the mice were sacrificed by carbon dioxide asphyxiation and the tumors were peeled off for further analysis. This study was conducted in strict accordance with the guidelines of the National Institutes of Health (NIH) on the use of experimental animals. Our protocol was approved by the Animal Experimental Ethics Committee of Nanjing Medical University.

### Immunohistochemical analysis

Tumor tissue samples were embedded in paraffin and cut into 4-mm-thick sections, which were stained with hematoxylin and eosin. For immunohistochemical studies, we incubated the samples overnight at 4 °C with anti-Ki67 antibodies against human targets. Thereafter, the sample was washed with PBS and the second antibody was added dropwise, following by incubation at 37 °C for 2 h in a water bath and then washing with PBS. After being treated with DAB solution and hematoxylin, the sections were washed with water and dehydrated, then treated with a clear medium and finally fixed on glass slides. The IHC staining was observed under a microscope to minimize subjective factors and to obtain the final synthesized result. When ≥50% of cancer cells were stained, the expression was considered positive.

### RNA sequencing analysis

RNA was isolated from BxPC-3 cells transfected with AGAP2-AS1 and from corresponding control cells. RNA preparation, sequencing, and library construction were performed using the BGISEQ-500 sequencing platform at the Beijing Genomics Institute (BGI). RNA was isolated from cell precipitation using TRIzol TM reagent (Invitrogen), and its quality was determined using a Bioanalyzer 2100 (Agilent). 23s and 16s rRNA were eliminated using a MicrobExpress kit (Ambion). Genomic DNA was removed by two digests using amplified-grade DNAse1 (Invitrogen). The RNA was cleaved and reverse-transcribed using random primers to obtain cDNA for library construction. The quality of the library was determined using the Bioanalyzer 2100. This library was then used for sequencing with the BGISEQ-500 (BGI). The original sequencing readings were filtered to remove connector readings, which have an unknown base of more than 10%, and poor-quality readings. The resulting clean read was stored in FASTQ format. Gene expression levels were quantified by the RSEM software package. The NOISeq method was used to screen for differentially expressed genes between two groups.

### Western blot analysis and antibodies

Transfected AsPC-1 and BxPC-3 cells were treated with an RIPA protein extraction reagent (Beyotime, Beijing, China) containing protease inhibitor and phenylmethylsulfonyl fluoride. After determining the protein concentration, approximately 50 μg of the protein extract was separated by 10% sodium dodecyl sulfate polyacrylamide gel electrophoresis and transferred to a nitrocellulose membrane (Sigma) with specific antibodies (Cell Signaling Technology, Boston, MA, USA). The intensities of the bands were observed and determined by densitometry (Quantity One software; Bio-Rad, Hercules, CA, USA), using GAPDH as a control.

### RNA immunoprecipitation

We performed the RIP experiments with the Magna RIP RBP immunoprecipitation kit (Millipore) in accordance with the manufacturer’s directions. AsPC-1 and BxPC-3 cells were lysed in complete RIP lysis buffer, and the cell extracts were then mixed with magnetic beads conjugated with specific antibodies or control IgG (Millipore) and incubated for 6 h at 4 °C. To remove the protein, the beads were incubated with proteinase K after washing. Finally, the purified RNA was subjected to qRT-PCR analysis. The EZH2 RIP assay antibody was from Abcam.

### Separation of cytoplasm and nuclear RNA

The nuclear and cytoplasmic fractions of AsPC-1 and BxPC-3 cells were separated using a PARIS kit (Life Technologies) in accordance with the manufacturer’s protocol. Reverse transcription and RT-PCR (SYBR Premix Ex Taq; TaKaRa) were performed with the extracted RNA. The primer sequences are described in Supplementary Table S1.

### Fluorescence in situ hybridization assays

BxPC-3 cells were fixed in 4% formaldehyde for 15 min and then washed with PBS. Pepsin (1% in 10 mmol/l HCl) was used to treat the fixed cells, followed by continuous dehydration with ethanol. The dried cells were mixed with 40 nmol/l of the FISH probe (U6, 18s, AGAP2-AS1 lncRNA) in a hybridization buffer and incubated at 80 °C for 2 min. After being left to stand at 55 °C for 2 h, the slides were washed and dehydrated, and finally observed and detected with Prolong Gold Antifade Reagent using DAPI. The RNA FISH probe was supplied by Ribobio.

### ChIP analysis

ChIP assays were performed using an EZ-ChIP kit according to the manufacturer’s instructions (Millipore). Immunoprecipitation was performed using anti-RREB1, anti-EZH2, and anti-H3K27me3 (Millipore) with the normal mouse IgG as a negative control. The primers were designed based on the promoter sequences of ANKRD1 and ANGPTL4, upstream of the ANKRD1 and ANGPTL4 gene transcription start sites (listed in the supplemental experimental procedures). These primers were then used for qRT-PCR according to the manufacturer’s instructions. Using the formula 2^[InputCt−TargetCt]^ × 0.1 × 100, the ChIP data were calculated as percentages with respect to the input DNA.

### Statistical analysis

We performed statistical analysis using the SPSS and GraphPad Prism 5 software. The significance of the differences between the experimental group and the control group was estimated by Student’s *t* test or chi-square test. The OS of PC patients was calculated using the Kaplan–Meier method and compared using a log-rank test. Pearson correlation coefficients were calculated using Prism 5. *P* < 0.05 was considered statistically significant.

## Supplementary information

Supplementary Table S1

Supplementary Table S2
