# Supplementary material for: RREB1-induced upregulation of the lncRNA AGAP2-AS1 regulates the proliferation and migration of pancreatic cancer partly through suppressing ANKRD1 and ANGPTL4
Source: Cell Death Dis. 2019 Feb 27;10(3):207. doi: 10.1038/s41419-019-1384-9 (PMC6393474; doi:10.1038/s41419-019-1384-9)
Supplement: Supplementary file 1 — Supplementary Table S1 [file 41419_2019_1384_MOESM1_ESM.docx]

| **Primers, siRNA and shRNA sequence** |  |
| --- | --- |
| **Primers** |  |
| GAPDH F  | GGGAGCCAAAAGGGTCAT |
| GAPDH R | GAGTCCTTCCACGATACCAA |
| AGAP2-AS1 F | TACCTTGACCTTGCTGCTCTC |
| AGAP2-AS1 R | TGTCCCTTAATGACCCCATCC |
| RREB1 F | GGGCTTATCCCCCAGTCAAA |
| RREB1 R | TCTCCGCATCCGACTGACT |
| ANKRD1 F | AGACACTTCTAGCCCACCCT |
| ANKRD1 R | AGCTCTGCCTCTCGTTGTTT |
| ANGPTL4 F | GTTTGCAGACTCAGCTCAAGG |
| ANGPTL4 R | CCAAGAGGTCTATCTGGCTCTG |
| EZH2 F | GAGCAAAGCTTACACTCCTTTCA |
| EZH2 R | ATAAGTGTTGGGTGTTGCATGA |
| U6 F | CTCGCTTCGGCAGCACA |
| U6 R | AACGCTTCACGAATTTGCGT |
| **promoter primers** |  |
| promoter-AGAP2-AS1 F | AACCCCAGTACCCCATCTTG |
| promoter-AGAP2-AS1 R | GGTAATTGGGGAGGGGAGAG |
| promoter-ANKRD1 F | TTAGCCCCAGACATCACTCC |
| promoter-ANKRD1 R | TGCCCTACATATCACACCCC |
| promoter-ANGPTL4 F | CAGGCTCAGAATGTTGGGGT |
| promoter-ANGPTL4 R | CATGCACTGAACGAAGGTGC |
| **siRNA sequence** | |
| si-AGAP2-AS1 1# | CCACTCCACCTCAAACTCTTACCTT |
| si-AGAP2-AS1 2# | GGGTCATTAAGGGACAGAGTTCAAG |
| si-AGAP2-AS1 3# | TCCGAGATGAAGCCTCAAA |
| si-RREB1 | CGCTGGCAGACATCCAGCAAAT |
| si-ANKRD1 | AAGTATGAAGCTGCTGTTACTT |
| si-ANGPTL4 | AGCAGAGTGGACTATTTGAAAT |
| si-EZH2 | CTCAGACGAGCTGATGAAGTAA |
| **shRNA sequence** | |
| sh-AGAP2-AS1 | GGATCCCCGGCCACTCCACCTCAAACTCTTACCTTCAAGAGAGGTAAGAGTTTGAGGTGGAGTGGTTTTTTAAGCTT |
