# Supplementary material for: RREB1-induced upregulation of the lncRNA AGAP2-AS1 regulates the proliferation and migration of pancreatic cancer partly through suppressing ANKRD1 and ANGPTL4
Source: Cell Death Dis. 2019 Feb 27;10(3):207. doi: 10.1038/s41419-019-1384-9 (PMC6393474; doi:10.1038/s41419-019-1384-9)
Supplement: Supplementary file 2 — Supplementary Table S2 [file 41419_2019_1384_MOESM2_ESM.pdf]

2530 putative sites were predicted with these settings (80%) in sequence named NC_000012.12:57724240-57726240

Model ID	Model name	Score	Relative score	Start	End	Strand	predicted site sequence
MA0073.1	RREB1	20.597	0.939155	1455	1474	1	CCCCAACCCACCCCCTGCCC
MA0073.1	RREB1	19.022	0.914711	1450	1469	1	CCCCACCCCAACCCACCCCC
MA0073.1	RREB1	15.246	0.856107	1449	1468	1	CCCCCACCCCAACCCACCCC
MA0073.1	RREB1	15.043	0.852956	1492	1511	1	CCCCAAAACGCCCTCCCCAC
MA0073.1	RREB1	13.802	0.833696	1439	1458	1	CCCGAGAGCACCCCCACCCC
MA0073.1	RREB1	13.369	0.826976	1454	1473	1	ACCCCAACCCACCCCCTGCC
MA0073.1	RREB1	12.941	0.820333	1448	1467	1	ACCCCCACCCCAACCCACCC
MA0073.1	RREB1	11.713	0.801274	1447	1466	1	CACCCCCACCCCAACCCACC

selectivity. In other words: while true functional will be detected in most cases, most predictions will correspond to sites bound in vitro but with no function in vivo. A number of additional contraints of the analysis can improve the prediction; phylogenetic footprinting is the most common. We recommend using the ConSite service, which uses the JASPAR datasets.
The review Nat Rev Genet. 2004 Apr;5(4):276-87 gives a comprehensive overview of transcription binding site prediction
